# Adenosquamous carcinoma of the conjunctiva: A case report

**DOI:** 10.3892/ol.2014.2008

**Published:** 2014-03-28

**Authors:** SATORU KASE, HIROSHI YOSHIKAWA, YUTAKA NAKAJIMA, MIKA NODA, SUSUMU ISHIDA

**Affiliations:** 1 Department of Ophthalmology, Hokkaido University Graduate School of Medicine, Sapporo, Hokkaido 060-8638, Japan; 2 Department of Ophthalmology, Graduate School of Medical Sciences, Kyushu University, Fukuoka 812-8582, Japan; 3 Division of Pathophysiological and Experimental Pathology, Department of Pathology, Graduate School of Medical Sciences, Kyushu University, Fukuoka 812-8582, Japan

**Keywords:** adenosquamous carcinoma, conjunctiva, histopathology

## Abstract

Adenosquamous carcinoma (ASC) is a rare form of malignancy which consists of two types of cell, including squamous cells and glandular-like cells. The current report presents the first known case of ASC in the conjunctiva and analyzes the histological findings. A 76-year-old female presented with right eyelid swelling in 2001. A right conjunctival tumor was noted and a biopsy was performed. Histologically, the tumor was diagnosed as a squamous cell carcinoma. The patient underwent radiotherapy, but the tumor rapidly relapsed. Subsequently, the patient underwent orbital exenteration. Histologically, the conjunctival tissues had been replaced with invasive tumor cells. A number of tumor cells demonstrated squamous differentiation with a keratinizing tendency, while other tumor cells exhibited mucin-producing activity with glandular formation. The conjunctival tumor was diagnosed as an ASC. At the time of writing, the patient is well without local recurrence or distant metastases. ASC typically exhibits aggressive biological behavior, and is associated with worse prognosis than conventional adenocarcinoma. Therefore, complete surgical excision is considered a key treatment for ASC of the conjunctiva.

## Introduction

Adenosquamous carcinoma (ASC) is a rare form of malignancy which consists of two types of cell, including squamous cells and glandular-like cells (1,2). It has been reported that ASC can be found as an isolated tumor in various systemic organs, including the stomach, intestines, uterus, lungs, esophagus, anus and vagina (3–8). However, ASC may also exist with other types of malignancy (3), which suggests that an accurate diagnosis is required in each case. Several studies have demonstrated that computed tomography scanning is particularly useful in the diagnosis of ASC (6,8), as radiographic findings differ between organs. ASC is known to show aggressive behavior in addition to metastatic spread (4). Imaoka *et al* recently analyzed clinical features and prognosis using a large number of pancreatic cancers including 28 cases with ASC, in which the median overall survival rate was unfavorable for ASC compared with that for ductal adenocarcinoma (7). Due to its aggressive nature, surgical excision is considered one of the major treatment options for ASC (4).

The conjunctiva contains columnar epithelium together with goblet cells, which tends to show squamous metaplasia in adults. Squamous cell carcinoma is one of the commonest conjunctival epithelial malignancies (9); however, ASC has yet to be reported. The present study reports the first known case of ASC in the conjunctiva and analyzes the histological findings.

## Case report

**Figure 1:**
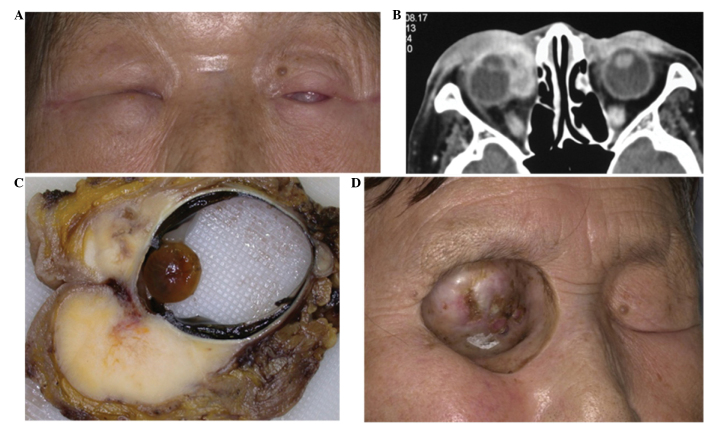
(A) Facial photograph and (B) computed tomography prior to treatment; (C) gross appearance of tumor following orbital exenteration; and (D) the patient following treatment.

**Figure 2:**
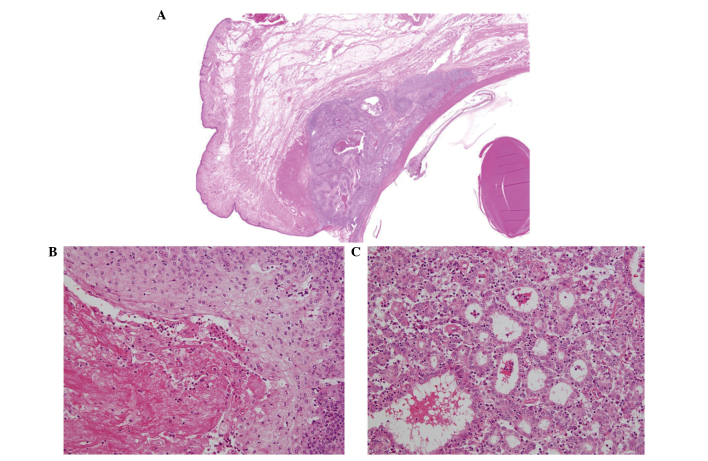
Histological findings in the excised tissue. (A) Tumor cells localized in the conjunctiva with intermingled cystic change (arrow); (B) tumor cells exhibit squamous differentiation with keratinizing tendency at high magnification; and (C) certain tumor cells exhibit mucin-producing activity and proliferate in tubular or solid patterns. Magnification: A, ×5; B, ×20; C, ×20.

A 76-year-old female presented with right eyelid swelling in 2001 (Fig. 1A). The patient had a medical history of conjunctival infection with *Chlamydia trachomatis* in both eyes. On initial clinical examination, a right conjunctival tumor was noted and a biopsy was performed. Histologically, the tumor was diagnosed as a squamous cell carcinoma. The patient underwent radiotherapy, but the tumor rapidly relapsed. Computed tomography demonstrated massive high-intensity eyelid and orbital mass lesions (Fig. 1B). The patient underwent orbital exenteration on March 7, 2003. The excised tissues revealed marked whitish nodules adjacent to the eyeball (Fig. 1C). Histologically, the excised tissue exhibited a collection of tumor cells localized in the conjunctiva with intermingled cystic change (Fig. 2A, arrows). At high magnification, the conjunctival tissues were replaced with invasive tumor cells. The majority of the tumor cells exhibited a high nucleus/cytoplasm ratio and severe nuclear atypia with frequent mitoses. Numerous tumor cells demonstrated squamous differentiation with a keratinizing tendency (Fig. 2B). Other tumor cells exhibited glandular formation (Fig. 2C). Tumor cells had invaded the tarsal plate, extraocular muscles and cornea. The conjunctival tumor was diagnosed as an ASC. The surgical margins were free of tumor cells and, at the time of writing, the patient is well without local recurrence or distant metastases (Fig. 1D).

## Discussion

The occurrence of ASC is more common in organs where adenocarcinomas arise frequently, including the stomach, intestines and uterus. ASC has also been identified in the esophagus, anus and vagina, where squamous cell carcinomas predominate (5). Squamous cell carcinoma is a common malignant epithelial tumor of the conjunctiva which occurs in predominantly male and immunosuppressed patients (9). Metastatic ASC involving the orbit has also been reported (10), but while it is known that primary ASC can arise from the lacrimal gland (11), there are no reports of ASC arising in the conjunctiva.

Although the pathogenesis of ASC remains largely unknown, the following four hypotheses have been proposed (5): i) Malignant transformation of both squamous and glandular-like cells originating from pleiotropic epithelial stem cells, ii) tumorigenesis of squamous metaplasia in the columnar epithelium, iii) transdifferentiation of adenocarcinoma to squamous cell carcinoma, and iv) the coexistence of both carcinomas. It is likely that the conjunctival epithelium can exhibit squamous metaplasia, from which squamous cell carcinoma arises. In addition, the conjunctiva contain microscopic pockets, called the crypts of Henle, around the eyeball which are responsible for secreting mucin, a proteinaceous substance that makes up the inner layer of tears (12). Therefore, the ASC in the present report may have arisen from malignant transformation of the crypts of Henle, including squamous and glandular cells. A second possibility is that a recurrent squamous cell carcinoma subsequently transformed into adenocarcinoma, since the patient had a medical history of eyelid squamous cell carcinoma.

ASC exhibits aggressive biological behavior, and is typically associated with worse prognosis than conventional adenocarcinoma. Therefore, surgical excision is considered a key treatment option for ASC. The extent of surgery is dependent on the location of the tumor (4); in a previous case, although surgical intervention was successfully conducted, the prognosis remained unfavorable (5). Thus, prognosis following surgical intervention is not always clear. In the present case, orbital exenteration was performed to eliminate the tumor cells completely. Histologically, the surgical margin was free of tumor cells, and the patient has remained well, without local recurrence or distant metastases, for 10 years. Additional observation is required to manage this rare aggressive tumor in the conjunctiva.
